# Part I: The Analytical Model Predicting Post-Yield Behavior of Concrete-Encased Steel Beams Considering Various Confinement Effects by Transverse Reinforcements and Steels

**DOI:** 10.3390/ma12142302

**Published:** 2019-07-18

**Authors:** Dinh Han Nguyen, Won-Kee Hong

**Affiliations:** Department of Architectural Engineering, Kyung Hee University, Yongin 17104, Korea

**Keywords:** double confining effects, steel beams encased by structural concrete, moment–curvature relationships, post-yield deflection, non-linear finite element analysis

## Abstract

The purpose of the work was to propose analytical model considering double confinements (provided by both transverse reinforcements and a wide flange steel section), which was verified by the nonlinear finite element analysis considering concrete-damaged plasticity. The scope of the effort and the procedures to achieve the aim of this study included the identification of the concrete confinements provided by both transverse reinforcements and a wide flange steel section based on the elasto-plastic model in tension for both rebar/steel sections and elasto-buckling for rebars in compression. The influence of rebar buckling in the compression zone on flexural moment strength was also investigated with and without considering confining effects offered by steel sections. The analytical approach predicted a post-yield behavior of composite beams based on the confining effect offered by both the shear reinforcement and wide steel flange sections. However, for beams without axial loads, the compressive zones with high and partial confinements for concrete sections at the yield and maximum load limit state were limited when compressive buckling failure was not considered, preventing the confining factors from significantly influencing the flexural load resisting capacity. An accurate flexural capacity of composite beams can be obtained when rebar was modeled with buckling in the compression zone.

## 1. Introduction

### 1.1. Literature Review

The use of concrete-encased steel beams requires an understanding of the individual merits of the two materials. Many tests have been performed to explore the post-yield behavior of the steel columns encased in structural concrete [1,2,3,4,5,6]. An analytical model that predicts the behavior of steel–concrete composite and hybrid structures were also developed by [7] in which the mechanical behavior of composite beams was described, under no restrictive assumptions on the connection and interaction. Stresses, strains, and displacements due to loads that induce elastic or inelastic behavior in the connection were predicted. The general analytical solution for the elastic three-layered plate with any interlayer (utterly compliant to relatively stiff) was also provided by [8]. Some of the structural applications where steel beams were confined by transverse reinforcements were presented by [9].

One of the studies of concrete-encased steel beams was carried out by [10], who predicted the axial compressive capacity of composite stub columns. More importantly, the emphasis of the previously mentioned modeling established the stress–strain relations for concrete confined by lateral reinforcement and by various structural steel sections of the composite columns. Strain compatibility analysis is used by the American Institute of Steel Construction [11,12] to design steel beams encased in structural concrete. Therein, four design procedures were suggested to predict the flexural strength of steel–concrete hybrid members. Researchers [13] presented a ‘modified strain compatibility approach’ for a broad range of yield and load limit states to calculate an accurate neutral axis, leading to the prediction of a nominal flexural capacity of the composite beams, which was not introduced in AISC 360-10 [11]. However, these approaches do not consider the confining effect provided by steel sections when predicting the nominal flexural capacity, leading to an inaccurate estimation of the actual compressive stress block. Analytical investigations of the inelastic behavior (particularly for understanding the concrete confinement effect by steel flanges encased in the concrete) are generally absent from the literature. Mander confinement models were used to model confined concrete by transverse reinforcements in this study. An additional confining effect provided by the wide flange of the steel section was divided into two zones, highly confined and partially confined regions, to calibrate the flexural strength with test data. 

### 1.2. Motivations and Objectives in This Study; Methodology for the Prediction of Nonlinear Structural Behavior of Steel–Concrete Composite Beams

The main goal of the present study was to analytically predict the post-yield moment–curvature relationships based on the double confining effects, which were subsequently compared with experimental results and nonlinear finite element analysis. This study focused on the strain compatibility-based simplified method. The material properties and stress–strain relationships included elasto-hardening and elasto-buckling of rebar steels to predict the post-yield behavior of the concrete-encased steel beams. 

Confinement factors for steel and concrete materials were identified by exploring the influence of wide flange of steel sections on the concrete confinement. The concrete zone confined by the steel flange was simplified to model confinement factors, K_h_ and K_p_, which were then validated by the test data and numerical results using non-linear finite element analysis considering concrete plasticity. In this model, rebars were assumed to buckle and lose their strength due to local buckling in the compression zone when the concrete cover reaches the peak strength. Buckling of rebar in the compression zone contributes to the formation of a hinge length and has an influence on the post-yield behavior. Simplified analytical equations based on strain compatibility were then proposed to estimate the equilibrium neutral axis depth and flexural moment strength. Mathematical expressions derived for the rapid evaluation of the flexural strength of the composite beams were implemented in Matlab for automated estimation of the post-yield behavior of the composite beams. The flexural load-carrying capacity and post-yield behavior were finally examined at the yield limit (the limit of elastic behavior and the beginning of plasticity), the maximum load limit state (the limit state at which the maximum loads are supported), and ultimate load limit state (the limit state at which the strength of the structures is terminated). The confining factors did not significantly influence the flexural load resisting capacity calculated based on strain compatibility. This was because the compressive zones at the yield and maximum load limit state were small, as shown in highly confined zones with concrete stress–strain profiles. However, a decrease in moment strengths was observed when rebars buckled during compression. Rebar buckling in the compression zone is not frequently considered in the flexural analysis of beams. 

## 2. Materials and Methods

### 2.1. Analytical Model of Concrete Confined by a Wide-Flange Steel Section

#### 2.1.1. A Confinement Effect Caused by the Structural Steel Sections

**Figure 1:**
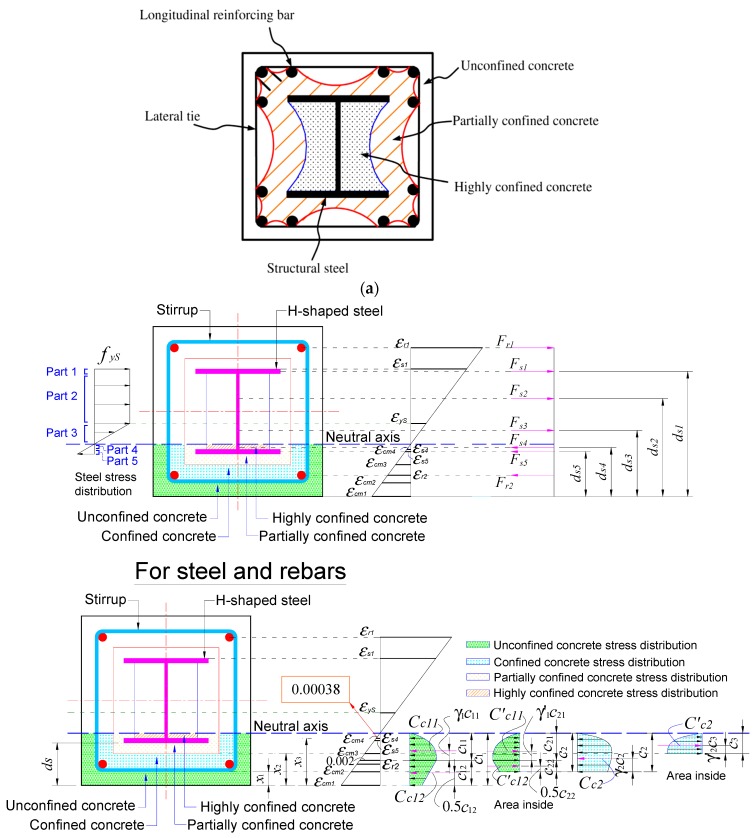

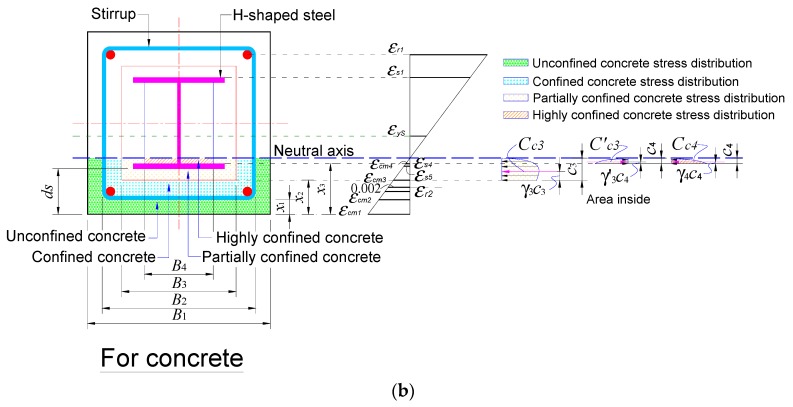
Four simplified zones with concrete stress–strain profiles. (**a**) Concrete confined by stirrups and a steel section (parabolic arching formed by the longitudinal bars and structural steel section [10]); (**b**) strains, stresses, and corresponding force components at the maximum limit state.

This study considered the confinement effect on concrete caused by lateral reinforcement and structural steel. It was assumed that parabolic arching of the area of the effectively confined concrete core occurred between the reinforcing bars in the cross section [14,15]. Similarly, researchers [10] assumed parabolic arching for concrete confined by the steel beam section, as presented in Figure 1a. The concrete encased steel section can be divided into three regions: (1) An unconfined concrete region outside the parabolic arch formed by the longitudinal bars, (2) a highly confined region inside the arch formed by the steel section, and (3) a partially confined region outside the highly confined concrete region and inside the parabolic arch formed by the longitudinal bars. In this study, these zones were simplified by four zones divided by straight lines, as shown in Figure 1b, in which concrete stress–strain distributions were demonstrated at the yield limit state. 

**Figure 2:**
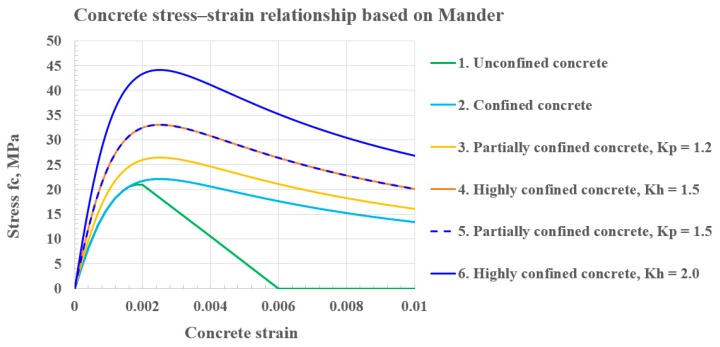
Concrete stress–strain relationships representing four zones (based on the confined Mander approach).

In this study, arching similar to that suggested by [4,5] was adopted to form the concrete region confined by the structural steel section. Researchers [10] defined the concrete strengths as *f_c_* = K_p_ *f_co_* and *f_cc_* = K_h_ *f_co_* in Equations (1) and (2), respectively, where K_p_ and K_h_ were defined as confinement factors for partially and highly confined concrete, respectively. Concrete stress–strain relationships representing the four zones, shown in Figure 2, were used in the proposed analytical models.

*f_cc_* = K_p_*f_co_* (1)

*f_cc_* = K_h_*f_co_* (2)

#### 2.1.2. Local Buckling of the Longitudinal Bars and the Structural Steel

**Figure 3:**
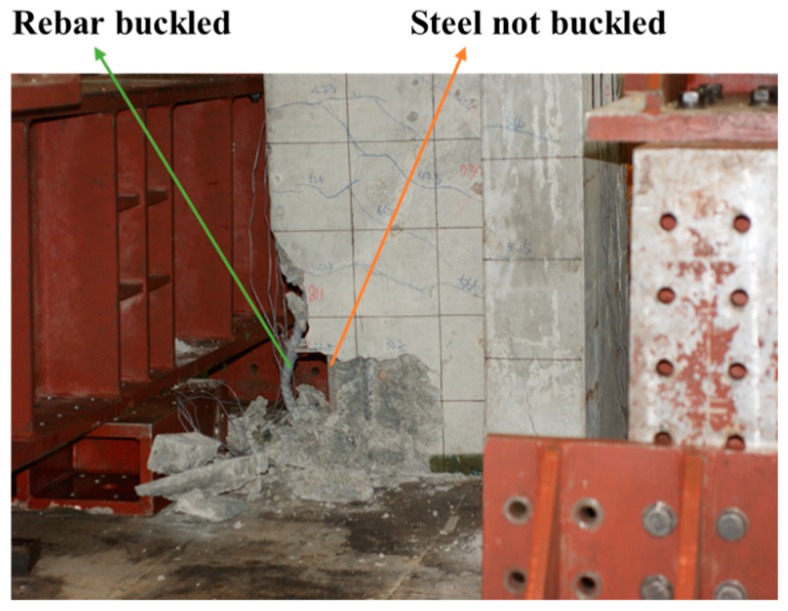
Rebars buckled during beam testing [17].

Researchers [16] suggested a constitutive model of longitudinal reinforcing rebars in the compression zone. In this study, this model was slightly modified by considering the confined concrete effect. The compressive rebar strength began to degrade to 20% of its yield strength from the maximum concrete strain (*ε_c,max_*), corresponding to the maximum confined concrete compressive strength (*f_cc_*), while maintaining a constant value after the longitudinal rebars under compression reached the concrete strain (*ε_cu_*). It was assumed that the spalling of the concrete cover caused the rebars to buckle and lose their strength. Figure 3 shows the local buckling of the longitudinal reinforcing rebars, which occurred after the partially confined concrete was crushed. The elasto-plastic constitutive model of longitudinal reinforcing steels in the tension zone was used to model the composite members subjected to a flexural bending moment. The constitutive relationship considering buckling was used for reinforcing steels only as it was observed in the experimental investigation, while no noticeable bucking of the steel section encased in concrete was found as shown in Figure 3. The elasto-plastic constitutive relationship was used for the compression and tension zone of the steel section.

### 2.2. Analytical Model of Confinement by Transverse Reinforcements and Wide Flange Steel Sections

#### 2.2.1. Strain Compatibility-Based Model

The steel beam encased in structural concrete considered with nomenclature describing the section is shown in Figure 1 and Appendix A. The neutral axes satisfying the equilibrium for yield limit states and maximum load limit state of the section are shown in Figure 1b based on four simplified zones with concrete stress–strain profiles. The analytical expressions for obtaining the neutral axis were derived at the limit state including the yield limit, maximum load, and ultimate load limit state. The steel flanges yielded at yield limit state. The maximum load limit state was the point when the nominal flexural strength was at the maximum value, while the ultimate flexural strength was obtained when the substantial contribution of the concrete was lost. The strain compatibility-based prediction of the precast composite beams was verified by the finite element analysis (FEA) study.

#### 2.2.2. At the Maximum Load Limit State

The concrete compressive forces for the four zones, including the unconfined concrete (green region), confined concrete (cyan region), partially confined concrete (yellow region), and highly confined concrete (orange region), are shown in Equations (3)–(11). Here, *c*_1_ is the neutral axis of the section at the maximum limit state. The mean stress factors *α* and centroid factors *γ* are derived in Equations (A1)–(A7) and (A8)–(A14) in Appendix A, respectively. The depths of the compressive concrete blocks are indicated by *c*_2_, *c*_3_, and *c*_4_, representing the zones confined by the steel section shown in Figure 1b. The relationships between the compressive concrete blocks and the neutral axis (*c*_1_) are also established in Equations (3)–(11). The Mander confining curve was used in these equations. For the unconfined concrete based on the Mander curve (green region of Figure 1), compressive forces due to the concrete block are given by:

(3) $$C_{c11}=\alpha_{1}\times c_{11}\times B_{1}\times \;f\prime_{c},$$

(4) $$C_{c12}=0.5\times c_{12}\times B_{1}\times \left(f\prime_{c}+f_{\varepsilon_{cm1}}\right),$$

(5) $$C\prime_{c11}=-\alpha^{\prime }{}_{1}\times c_{21}\times B_{2}\times {f^{\prime }}_{c},$$

(6) $$C\prime_{c12}=-0.5\times c_{22}\times B_{2}\times \left({f^{\prime }}_{c}+f_{\varepsilon_{cm1}}\right).$$

For confined concrete based on the Mander curves (cyan region of Figure 1),

(7) $$C_{c2}=\alpha_{2}\times c_{2}\times B_{2}\times \;f\prime_{cc},$$

(8) $$C\prime_{c2}=-{\alpha \prime }_{2}\times c_{3}\times B_{3}\times \;f\prime_{cc}.$$

For partially confined concrete based on the Mander curves (yellow region of Figure 1),

(9) $$C_{c3}=\alpha_{3}\times c_{3}\times B_{3}\times K_{p}\times f\prime_{cc},$$

(10) $$C\prime_{c3}=-{\alpha \prime }_{3}\times c_{4}\times B_{4}\times K_{p}\times f\prime_{cc}.$$

For the highly confined concrete, based on the Mander approach (orange region of Figure 1),

(11) $$C_{c4}=\alpha_{4}\times c_{4}\times B_{4}\times K_{h}\times f\prime_{cc},$$

where the depths of each compressive concrete block were obtained based on knowledge of the neutral axis, $c_{1}.\;$The compressive concrete blocks $c_{2},\;c_{3},\;c_{4},$ the mean stress factors $\alpha_{i}$, and the centroid factors $\gamma_{i}$ are shown in the Appendix A.

The equilibrium equations are given in Equations (12) and (13), where the neutral axis, $c_{1},$ is calculated.

(12) $$F_{axial}\left(\mathrm{zero}\;\mathrm{when}\;\mathrm{there}\;\mathrm{is}\;\mathrm{no}\;\mathrm{axial}\;\mathrm{loads}\right)+C_{c}+F_{R_{compression}}+F_{steel_{compression}}=\;F_{R_{tension}}+F_{steel_{tenstion}}$$

or

(13) $$F_{axial}=F_{R_{tension}}+F_{steel_{tenstion}}-C_{c}-F_{R_{compression}}-F_{steel_{compression}}$$

where the internal forces contributed by the structural components of the section are shown as follows:

(14) $$F_{R_{tension}}=\;A_{r1}\times f_{yR}$$

(15) $$F_{steel_{tenstion}}=\;\left(A_{s1}+A_{s2}+0.5A_{s3}\right)\times f_{yS}$$

where $A_{s1}=b_{f}\times t_{f1},A_{s2}=\left(d_{s}+h-c_{1}-t_{f1}\right)\times t_{w},\;\;A_{s3}=\left(d_{s}+h-c_{1}-t_{f1}\right)\times t_{w}$.

Equation (15) can be rewritten as below,

(16) $$F_{steel_{tenstion}}=\;\left(b_{f}\times t_{f1}+\left(d_{s}+h-c_{1}-t_{f1}\right)\times t_{w}+0.5\left(d_{s}+h-c_{1}-t_{f1}\right)\times t_{w}\right)\times f_{yS}$$

(17) $$C_{c}=\alpha_{1}\times c_{11}\times B_{1}\times {f^{\prime }}_{c}+0.5\times c_{12}\times B_{1}\times \left({f^{\prime }}_{c}+f_{\varepsilon_{cm1}}\right){\alpha^{\prime }}_{1}\times c_{21}\times B_{2}\times {f^{\prime }}_{c}\;-0.5c_{22}\times B_{2}\times \left({f^{\prime }}_{c}+f_{\varepsilon_{cm2}}\right)+\alpha_{2}\times c_{2}\times B_{2}\times {f^{\prime }}_{cc}\;-{\alpha^{\prime }}_{2}\times c_{3}\times B_{3}\times {f^{\prime }}_{cc}+\alpha_{3}\times c_{3}\times B_{3}\times K_{p}\times {f^{\prime }}_{cc}\;-{\alpha^{\prime }}_{3}\times c_{4}\times B_{4}\times K_{p}\times {f^{\prime }}_{cc}+\alpha_{4}\times c_{4}\times B_{4}\times K_{h}\times {f^{\prime }}_{cc}$$

(18) $$F_{R_{compression}}=A_{r2}\times E_{r}\times \varepsilon_{s1}\times \frac{c_{1}-d_{2}}{d_{s}+h-c_{1}}$$

where the tensile strain of $\varepsilon_{s1}$ at steel flange was obtained as 0.0055.

(19) $$F_{steel_{compression}}=0.5\times A_{s4}\times E_{s}\times \varepsilon_{s1}\times \frac{c_{1}-\left(d_{s}+t_{f2}\right)}{d_{s}+h-c_{1}}\;+\;A_{s5}\times E_{s}\times \varepsilon_{s1}\;\times \;\frac{c_{1}-\left(d_{s}+0.5t_{f2}\right)}{d_{s}+h-c_{1}},$$

where $A_{s4}=\left[c_{1}-\left(d_{s}+t_{f2}\right)\right]\times t_{w},\;A_{s5}=b_{f}\times t_{f2}.$

Equation (19) can be rewritten as below,

(20) $$F_{steel_{compression}}=0.5\times \left[c_{1}-\left(d_{s}+t_{f2}\right)\right]\times t_{w}\times E_{s}\times \varepsilon_{s1}\times \frac{c_{1}-\left(d_{s}+t_{f2}\right)}{d_{s}+h-c_{1}}\;+\;b_{f}\times t_{f2}\times E_{s}\times \varepsilon_{s1}\;\times \;\frac{c_{1}-\left(d_{s}+0.5t_{f2}\right)}{d_{s}+h-c_{1}}$$

Equation (13) can be expressed as follows:

(21) $$F_{axial}=F_{R_{tension}}+F_{steel_{tenstion}}-C_{c}-F_{R_{compression}}-F_{steel_{compression}}=\;\left\{A_{r1}\times f_{yR}\right\}+\;\left\{\left(A_{s1}+A_{s2}+0.5A_{s3}\right)\times f_{yS}\right\}-\;\{\alpha_{1}\times c_{11}\times B_{1}\times {f^{\prime }}_{c}+\;0.5\times c_{12}\times B_{1}\times \left({f^{\prime }}_{c}+f_{\varepsilon_{cm1}}\right)-{\alpha^{\prime }}_{1}\times c_{21}\times B_{2}\times {f^{\prime }}_{c}-0.5c_{22}\times B_{2}\times \left({f^{\prime }}_{c}+f_{\varepsilon_{cm2}}\right)\;+\alpha_{2}\times c_{2}\times B_{2}\times {f^{\prime }}_{cc}-{\alpha^{\prime }}_{2}\times c_{3}\times B_{3}\times {f^{\prime }}_{cc}+\alpha_{3}\times c_{3}\times B_{3}\times K_{p}\times {f^{\prime }}_{cc}-{\alpha^{\prime }}_{3}\times c_{4}\times B_{4}\times K_{p}\times {f^{\prime }}_{cc}+\alpha_{4}\times c_{4}\times B_{4}\times K_{h}\times {f^{\prime }}_{cc}\}\;-\;\left\{A_{r2}\times E_{r}\times \varepsilon_{s1}\times \frac{c_{1}-d_{2}}{d_{s}+h-c_{1}}\right\}-\;\left\{0.5\times A_{s4}\times E_{s}\times \varepsilon_{s1}\times \frac{c_{1}-\left(d_{s}+t_{f2}\right)}{d_{s}+h-c_{1}}\;+\;A_{s5}\times E_{s}\times \varepsilon_{s1}\times \frac{c_{1}-\left(d_{s}+0.5t_{f2}\right)}{d_{s}+h-c_{1}}\right\}=\;\left\{A_{r1}\times f_{yR}\right\}+\left\{\left(b_{f}\times t_{f1}+\left(d_{s}+h-c_{1}-t_{f1}\right)\times t_{w}+0.5\left(d_{s}+h-c_{1}-t_{f1}\right)\times t_{w}\right)\times f_{yS}\right\}\;-\;\{\alpha_{1}\times c_{11}\times B_{1}\times {f^{\prime }}_{c}+0.5\times c_{12}\times B_{1}\times \left({f^{\prime }}_{c}+f_{\varepsilon_{cm1}}\right)-{\alpha^{\prime }}_{1}\times c_{21}\times B_{2}\times {f^{\prime }}_{c}\;\;-0.5c_{22}\times B_{2}\times \left({f^{\prime }}_{c}+f_{\varepsilon_{cm2}}\right)+\alpha_{2}\times c_{2}\times B_{2}\times {f^{\prime }}_{cc}-\;{\alpha^{\prime }}_{2}\times c_{3}\times B_{3}\times {f^{\prime }}_{cc}+\alpha_{3}\times c_{3}\times B_{3}\times K_{p}\times {f^{\prime }}_{cc}-{\alpha^{\prime }}_{3}\times c_{4}\times B_{4}\times K_{p}\times {f^{\prime }}_{cc}+\;\alpha_{4}\times c_{4}\times B_{4}\times K_{h}\times {f^{\prime }}_{cc}\;\}-\;\left\{A_{r2}\times E_{r}\times \varepsilon_{s1}\times \frac{c_{1}-d_{2}}{d_{s}+h-c_{1}}\right\}-\;\{0.5\times \left[c_{1}-\left(d_{s}+t_{f2}\right)\right]\times t_{w}\times E_{s}\times \varepsilon_{s1}\times \frac{c_{1}-\left(d_{s}+t_{f2}\right)}{d_{s}+h-c_{1}}\;+\;b_{f}\times t_{f2}\times E_{s}\times \varepsilon_{s1}\times \frac{c_{1}-\left(d_{s}+0.5t_{f2}\right)}{d_{s}+h-c_{1}}\}$$

The nominal moment strength at the yield limit state was then obtained using Equation (22)

(22) $$M_{nominal}{}_{\;}=M_{R/centroid}+M_{steel/centroid}-M_{Conc/centroid},$$

where the flexural moment capacities provided by the structural components (with respect to the centroid) are shown as follows:

(23) $$M_{R/centroid}=\;A_{r1}\times f_{yR}\times (d_{1}-d_{c})-A_{r2}\times E_{r}\times \varepsilon_{s1}\times \frac{c_{1}-d_{2}}{d_{s}+h-c_{1}}\times (d_{2}-d_{c})$$

(24) $$M_{steel/centroid}=\;\left(A_{s1}\times f_{yS}\times \left(d_{s1}-d_{c}\right)\right)+\left(A_{s2}\times f_{yS}\times \left(d_{s2}-d_{c}\right)\right)\;+\left(0.5A_{s3}\times f_{yS}\times \left(d_{s3}-d_{c}\right)\right)\;-0.5A_{s4}\times E_{s}\times \varepsilon_{s1}\times \frac{c_{1}-\left(d_{s}+t_{f2}\right)}{d_{s}+h-c_{1}}\times \left(d_{s4}-d_{c}\right)\;-A_{s5}\times E_{s}\times \varepsilon_{s1}\times \frac{c_{1}-\left(d_{s}+0.5t_{f2}\right)}{d_{s}+h-c_{1}}\times \left(d_{s5}-d_{c}\right)$$

or

(25) $$M_{steel/centroid}=\;\left(b_{f}\times t_{f1}\times f_{yS}\times \left(d_{s1}-d_{c}\right)\right)\;+\left(\left(d_{s}+h-c_{1}-t_{f1}\right)\times t_{w}\times f_{yS}\times \left(d_{s2}-d_{c}\right)\right)\;+\left(0.5\left(d_{s}+h-c_{1}-t_{f1}\right)\times t_{w}\times f_{yS}\times \left(d_{s3}-d_{c}\right)\right)\;-0.5\left[c_{1}-\left(d_{s}+t_{f2}\right)\right]\times t_{w}\times E_{s}\times \varepsilon_{s1}\times \frac{c_{1}-\left(d_{s}+t_{f2}\right)}{d_{s}+h-c_{1}}\times \left(d_{s4}-d_{c}\right)\;-b_{f}\times t_{f2}\times E_{s}\times \varepsilon_{s1}\times \frac{c_{1}-\left(d_{s}+0.5t_{f2}\right)}{d_{s}+h-c_{1}}\times \left(d_{s5}-d_{c}\right)$$

(26) $$M_{Conc/centroid}=[\alpha_{1}\times c_{11}\times B_{1}\times {f^{\prime }}_{c}\times \left(\gamma_{1}\times c_{11}+c_{12}-d_{c}\right)\;+0.5\times c_{12}\times B_{1}\times \left(f{’}_{c}+f_{\varepsilon_{cm1}}\right)\times \left(0.5c_{12}-d_{c}\right)\;-{\alpha^{\prime }}_{1}\times c_{21}\times B_{2}\times {f^{\prime }}_{c}\times \left({\gamma^{\prime }}_{1}\times c_{21}+c_{22}+x_{1}-d_{c}\right)\;-0.5c_{22}\times B_{2}\times \left({f^{\prime }}_{c}+f_{\varepsilon_{cm2}}\right)\times \left(0.5c_{22}+x_{1}-d_{c}\right)]\;+[\alpha_{2}\times c_{2}\times B_{2}\times {f^{\prime }}_{cc}\times \left(\gamma_{2}\times c_{2}+x_{1}-d_{c}\right)\;-{\alpha^{\prime }}_{2}\times c_{3}\times B_{3}\times {f^{\prime }}_{cc}\times \left({\gamma^{\prime }}_{2}\times c_{3}+x_{2}-d_{c}\right)]\;+[\alpha_{3}\times c_{3}\times B_{3}\times K_{p}\times {f^{\prime }}_{cc}\times \left(\gamma_{3}\times c_{3}+x_{2}-d_{c}\right)\;-{\alpha^{\prime }}_{3}\times c_{4}\times B_{4}\times K_{p}\times {f^{\prime }}_{cc}\times \left({\gamma^{\prime }}_{3}\times c_{4}+x_{3}-d_{c})\right]\;+\left[\alpha_{4}\times c_{4}\times B_{4}\times K_{h}\times {f^{\prime }}_{cc}\times \left(\gamma_{4}\times c_{4}+x_{3}-d_{c}\right)\right]$$

where the lever arms for the moment calculations are given as follows:

$d_{s1}=d_{s}+h-0.5t_{f1}$
$d_{s2}=0.5\left(d_{s}+h-c_{1}\right)\times \left(1+\frac{\varepsilon_{yS}}{\varepsilon_{s1}}\right)-0.5t_{f1}+c_{1}$
$d_{s3}=\frac{2}{3}\times \frac{\varepsilon_{yS}}{\varepsilon_{s1}}\left(d_{s}+h-c_{1}\right)+c_{1}$
$d_{s4}=\frac{1}{3}\left(c_{1}+2d_{s}+2t_{f2}\right)$
$d_{s5}=d_{s}+0.5t_{f2}$.

Equation (22) can be rewritten as follows:

(27) $$M_{nominal}=\;M_{R/centroid}+M_{steel/centroid}-M_{Conc/centroid}\;=\left\{A_{r1}\times f_{yR}\times (d_{1}-d_{c})-A_{r2}\times E_{r}\times \varepsilon_{s1}\times \frac{c_{1}-d_{2}}{d_{s}+h-c_{1}}\times (d_{2}-d_{c})\right\}+\;\{\left(b_{f}\times t_{f1}\times f_{yS}\times \left(d_{s}+h-0.5t_{f1}-d_{c}\right)\right)+\;\left(\left(d_{s}+h-c_{1}-t_{f1}\right)\times t_{w}\times f_{yS}\times \left(0.5\left(d_{s}+h-c_{1}\right)\times \left(1+\frac{\varepsilon_{yS}}{\varepsilon_{s1}}\right)-0.5t_{f1}+c_{1}-d_{c}\right)\right)\;+\left(0.5\left(d_{s}+h-c_{1}-t_{f1}\right)\times t_{w}\times f_{yS}\times \left(\frac{2}{3}\times \frac{\varepsilon_{yS}}{\varepsilon_{s1}}\left(d_{s}+h-c_{1}\right)+c_{1}-d_{c}\right)\right)\;-0.5\left[c_{1}-\left(d_{s}+t_{f2}\right)\right]\times t_{w}\times E_{s}\times \varepsilon_{s1}\times \frac{c_{1}-\left(d_{s}+t_{f2}\right)}{d_{s}+h-c_{1}}\times \left(\frac{1}{3}\left(c_{1}+2d_{s}+2t_{f2}\right)-d_{c}\right)\;-b_{f}\times t_{f2}\times E_{s}\times \varepsilon_{s1}\times \frac{c_{1}-\left(d_{s}+0.5t_{f2}\right)}{d_{s}+h-c_{1}}\times \left(d_{s}+0.5t_{f2}-d_{c}\right)\}\;-\{(\alpha_{1}\times c_{11}\times B_{1}\times {f^{\prime }}_{c}\times \left(\gamma_{1}\times c_{11}+c_{12}-d_{c}\right)+0.5\times c_{12}\times B_{1}\times \left(f{’}_{c}+f_{\varepsilon_{cm1}}\right)\times \;\times \left(0.5c_{12}-d_{c}\right)-{\alpha^{\prime }}_{1}\times c_{21}\times B_{2}\times {f^{\prime }}_{c}\times \left({\gamma^{\prime }}_{1}\times c_{21}+c_{22}+x_{1}-d_{c}\right)-0.5c_{22}\times B_{2}\times \left({f^{\prime }}_{c}+f_{\varepsilon_{cm2}}\right)\;\times (0.5c_{22}+x_{1}-d_{c}))+(\alpha_{2}\times c_{2}\times B_{2}\times {f^{\prime }}_{cc}\times (\gamma_{2}\times c_{2}+x_{1}-d_{c})-{\alpha^{\prime }}_{2}\times c_{3}\times B_{3}\times {f^{\prime }}_{cc}\;\times ({\gamma^{\prime }}_{2}\times c_{3}+x_{2}-d_{c}))+(\alpha_{3}\times c_{3}+x_{3}\times B_{3}\times K_{p}\times {f^{\prime }}_{cc}\times (\gamma_{3}\times c_{3}+x_{2}-d_{c})\;-{\alpha^{\prime }}_{3}\times c_{4}\times B_{4}\times K_{p}\times {f^{\prime }}_{cc}\times ({\gamma^{\prime }}_{3}\times c_{4}+x_{3}-d_{c}))+(\alpha_{4}\times c_{4}\times B_{4}\times K_{h}\times {f^{\prime }}_{cc}\times (\gamma_{4}\times c_{4}+x_{3}-d_{c}))\}$$

#### 2.2.3. Validation of Analytical Model Based on Non-Linear Finite Element Analysis

**Figure 4:**
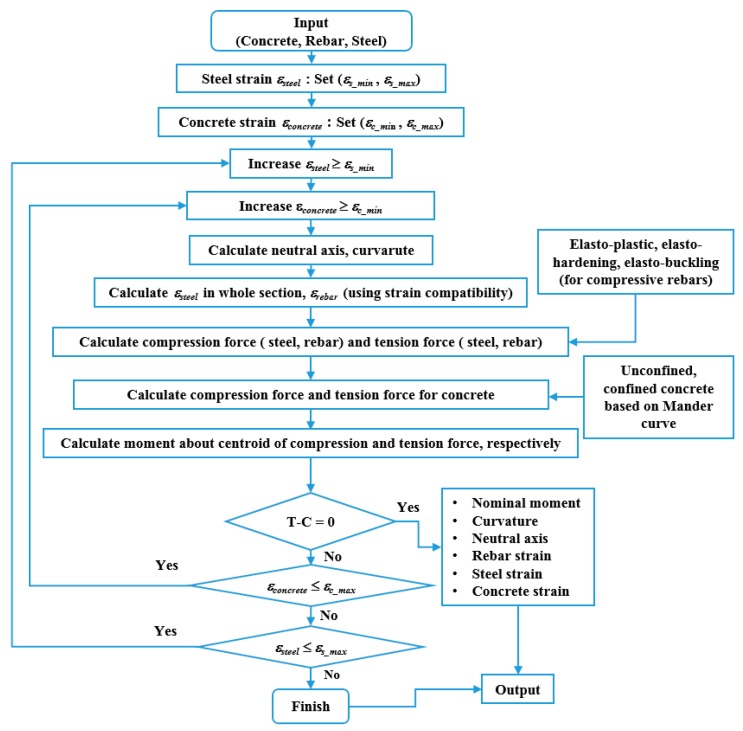
Computing algorithm based on strain compatibility.

The steel section (H-250 × 250 × 9 × 14) with a yield strength of 350 MPa and reinforcing steels (4-HD25) with yield strengths of 550 MPa were used in the analysis. Both have a Young’s modulus of 200,000 MPa. The concrete encasing steel section and rebars had compressive and tensile strengths of 21 MPa and 2.1 MPa, respectively, both with a Young’s modulus of 21,538 MPa. The yield strengths of the H-steel, reinforcing bars, and the concrete compressive strength were obtained from test samples. In Figure 1b, the compressive concrete strains at extreme fiber and at steel flange were found as 0.00384 and 0.00038, respectively, at maximum limit state. Figure 4 shows the computing algorithm used to estimate the neutral axis and the corresponding nominal moment capacity of the steel–concrete composite beam section when axial loads are not applied. The algorithm automatically calculates neutral axes for a total of 1200 strains at extreme fiber of the upper section, which corresponded to the same numbers of strains at the lower extreme fiber, performing 1,440,000 iterations to locate the neutral axis of the entire cross sections of the composite beams from top to bottom. The neutral axes of the composite sections were efficiently and accurately identified by finding a location satisfying equilibrium equations. The algorithm also calculated parameters required to design composite beams including the nominal moment capacity, based on the section configurations and material properties. Results were verified using nonlinear finite element analysis considering concrete plasticity, as can be seen in Results and Discussion which summarizes the nominal moment capacities at the yield, maximum load, and ultimate load limit state, showing the influence of the confined concrete effect caused by the steel section for the various confining factors, including K_h_ and K_p_.

### 2.3. Finite Element Analysis of the Composite Beams

#### 2.3.1. Material Properties and Parameters

**Figure 5:**
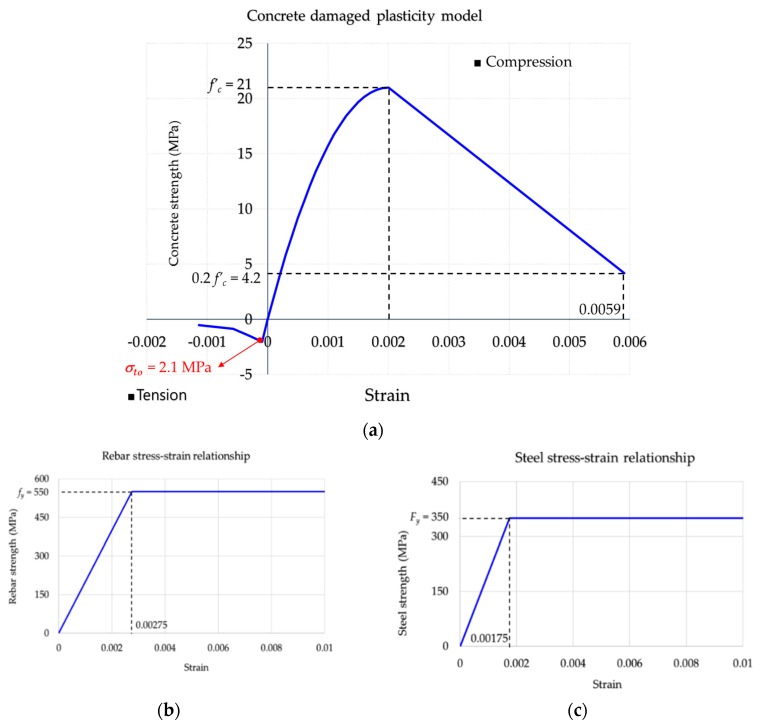
Materials model using in ABAQUS: (**a**) Concrete material model; (**b**) rebar material model; (**c**) steel material model.

In the FEA model, concrete damaged plasticity model for the behavior of concrete and the elasto-plasticity model for rebar and steel sections were considered. The stress–strain relationships for these materials are shown in Figure 5.

Researchers [18] suggested the use of a dilation angle of 30° for reinforced concrete beams. The dilatation angle ψ employed in the calibration of the FE model was introduced based on the non-associated Druker–Prager formulation, and it can be expressed as follows:

(28) $$G\left(\sigma \right)=\sqrt{{\left(\epsilon \sigma_{t0}tan\psi \right)}^{2}+{\bar{q}}^{2}}-\bar{p}tan\psi .$$

Here, ε denotes the eccentricity, which was set as 0.1. The importance of eccentricity in Equation (28) was to provide the rate at which the asymptote for the plastic potential function was evaluated, while the term $\sigma_{t0}$ represents the uniaxial tensile stress. The FEA parameters for the concrete material used to model damaged plasticity included *f_bo_/f_co_, K*, and viscosity, which were defined to be 1.16, 0.6667, and 0.001, respectively.

#### 2.3.2. Element Descriptions

**Figure 6:**
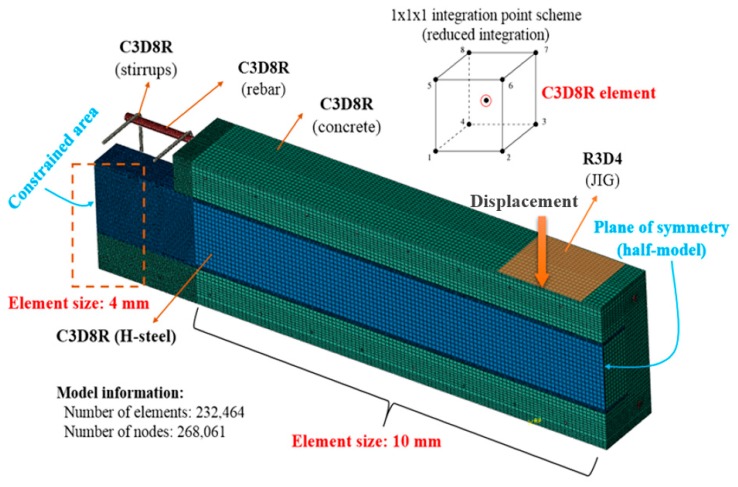
Elements selected for the FE model.

The elements of type C3D8R were chosen to represent the structural behavior of the steel–concrete composite beams, as shown in Figure 6. A fine mesh of 4 mm was assigned at the beam fixed end, while the remaining part of the beams was discretized with a coarse mesh of 10 mm. There were 268,061 total elements recorded in the FE model.

#### 2.3.3. Modeling of Reinforcing Bars and H-Steels for Composite Beams

**Figure 7:**
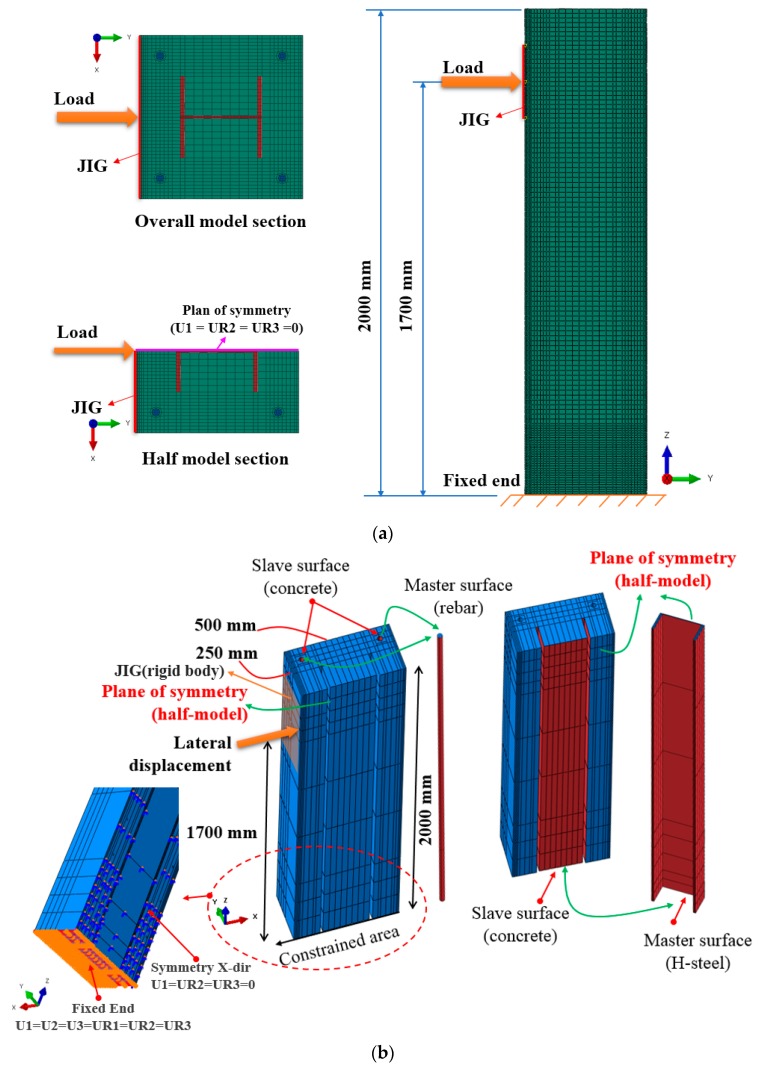
Finite element analysis (FEA) model for verification: (**a**) Overall model with load application; (**b**) definition of interactions: H-steel, reinforcing bars, and concrete.

The definition of interactions between the reinforcing bars, steel sections, and the concrete is an important issue for modeling steel–concrete composite beams. Previous studies adopted the embedded method to model the bond behavior between the concrete and reinforcements [19,20,21,22,23]. This method allows ABAQUS users to place the embedded elements (reinforcing bars and steel sections) into the host elements (concrete). ABAQUS tracks the embedded elements, which are then constrained by the response of the host elements. The translational DOFs of the nodes are eliminated in cases when the embedded elements lie within the host region; these nodes are referred to as embedded nodes. The translational movements of embedded elements are controlled by the host elements. Although embedding reinforcing bars into the concrete can be an easy and straightforward task, this method cannot properly simulate the real behavior of elements lying within the host elements. It is almost impossible for reinforcing bars to experience the necking failure mechanism when they are embedded into the concrete. In the present study, reinforcing bars and H-steels were tied to the concrete surface using the tie contact model, which is available in ABAQUS. The overall FEA model is shown in Figure 7a whereas the two surfaces, i.e., master and slave surfaces, were selected as illustrated in Figure 7b. The surfaces of the H-steels and reinforcing bars were defined as master surfaces, while the concrete surface was designated as a slave surface. The assigned tie constraint method fuses together the master and slave surfaces so that relative motion between the two surfaces cannot occur. The rotations between contacts, however, were permitted with buckling of the embedded elements. 

A rigid body object (JIG) was used with the dimensions 300 mm × 500 mm to transfer the concentrated load to the specimen, preventing the local failure of a single node in the FEA model, and relieving stress concentration. The FEA results were compared with test data to verify the reliability of the proposed tie modeling technique. It was found that the FE models constructed using the proposed modeling technique accurately predicted the post-yield behavior of the tested steel–concrete composite beam.

**Figure 8:**
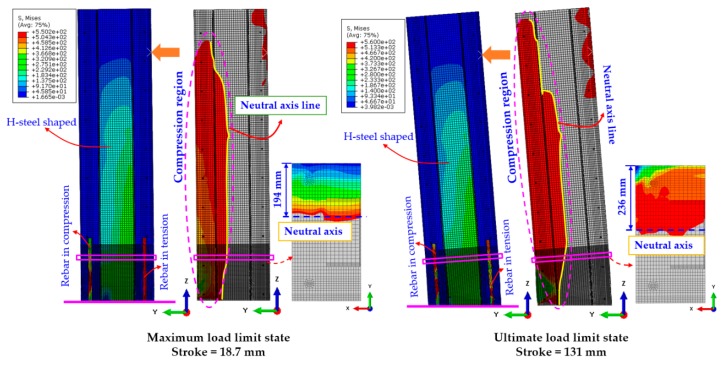
Stress status at maximum and the ultimate limit state.

The stress status maximum and ultimate limit state were demonstrated in the Figure 8. The deflections and the compression region of the specimen were also shown at those limit states. The compression region (illustrated by red color) and the tension regions are separated by the neutral axes (indicated by the yellow color) along the length of the beam. At the maximum and ultimate limit state, the strokes are of 18.7 mm and 131 mm and the neural axes of 194 mm and 236 mm were calculated based on the tie model, respectively.

**Figure 9:**
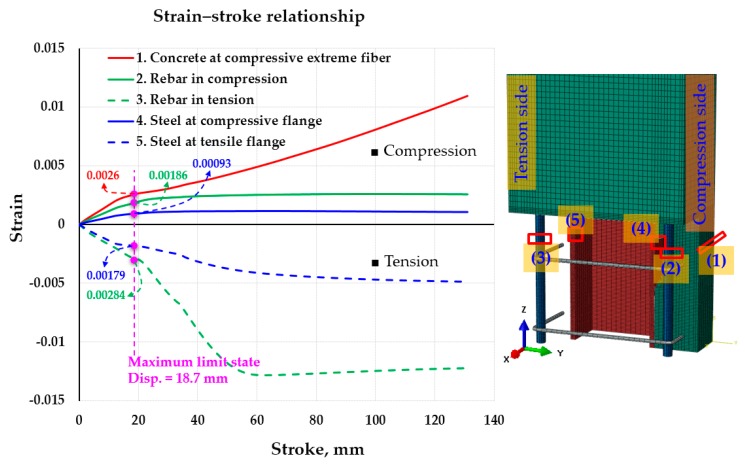
Strain vs. stroke curve for the components of the composite beam (concrete, rebar, H-steel).

In Figure 9, the relationships of the strains of beam elements (concrete, rebar, and steel) and the displacements are demonstrated. The strains corresponding to concrete at compressive extreme fiber (location (1)), rebar in compression (location (2)), rebar in tension (location (3)), steel at compressive flange (location (4)), and steel at tensile flange were indicated by the Legends 1 to 5. All strains increased rapidly up to the maximum limit state with a displacement of 18.7 mm, then increased slowly, except for the rebar in tension. At maximum limit state, the concrete at extreme fiber attained a strain of 0.0026 whereas rebar in compression, steel at the compressive flange, steel at the tensile flange, and rebar in tension reached the strains of 0.00186, 0.00093, 0.00179, and 0.00284, respectively. In this limit state, the re-bars and the H-steel shaped flange in tension started to yield with the strains of 0.00284 and 0.00179 (re-bar yield strain $\varepsilon_{yR}=f_{yR}/E_{s}\;=550/200000=0.00275$; steel yield strain $\varepsilon_{yS}=f_{yS}/E_{s}\;=350/200000=0.00175$). Thereafter, the strains of rebar in compression and steel at the compressive flange reached the maximum of 0.0025 and 0.0011 at the stroke of 27 mm; they kept these values until the ultimate load limit state with a stroke of 131 mm creating the long flat top regions. The concrete strain and the steel strain in the tensile flange increased gradually from 0.0026 (the maximum limit state) to 0.011 (ultimate load limit state) and from 0.00179 (the maximum limit state) to 0.005 (ultimate load limit state), respectively. Besides, the rebar strain in tension attained the maximum strain of 0.0128 at the stroke of 62.9 mm, after which strains gradually decreased to the strain of 0.0122 at the end of the test (stroke of 131 mm).

#### 2.3.4. Non-Linear Finite Element Analysis Based on Concrete Plasticity

**Figure 10:**
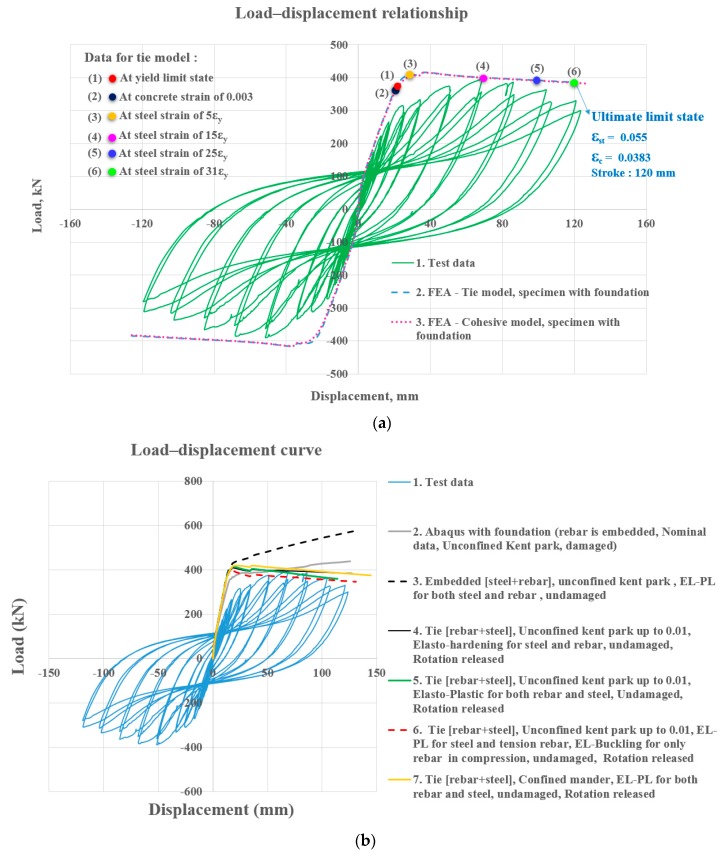
Finite element analysis compared with test data [25] (with base vs. without base): (**a**) With base rotation; test data included base rotation; (**b**) without base rotation (FEA); test data included base rotation.

The authors obtained test data of Figure 10 in the previous study [24] and found numerical data that matched the test data via finite element analysis based on the tie model established between the two separate surfaces. A numerical investigation of the proposed frames using finite element analysis (FEA) based on concrete plasticity was carried to explore the nonlinear structural behavior of the section. The FEA parameters identified to best describe the test data were obtained from the earlier study of the authors [25]. The numerical model of composite beams at fixed base was established to explore the post-yield deflection of composite beams. The surfaces between the concrete and rebars, and between concrete and the steel section were considered to be tied. The tie model ensured perfect structural bonds, allowing no relative motion between the materials, whereas buckling or necking of the rebars was allowed. In these tie models, the rotations were released. Once the FEA model with rotational effect was calibrated with the test data as shown in Figure 10a, the foundation was removed from the model by performing another non-linear finite element analysis as shown in Figure 10b, which was undisturbed by the rotational components due to the foundation. The numerical model of composite beams at fixed base was then established to explore the post-yield deflection of composite beams. In Figure 10b, the load–displacement relationships represented by Legend 2 were obtained based on the fixed base. It was obvious that they differ from those observed from the test data because test data contained rotational components. The flexural strength of the sections represented by Legends 4 to 6 were compared using various constitutive relationships of rebar and steel sections. The strength represented by Legend 6 was predicted by the model with elasto-buckling constitutive relationships for the rebar sections in compression, which slightly underestimated the flexural strength. However, the strength shown in Legend 4 was slightly overestimated when the elasto-hardening rebars and steel section were used. The closest comparison with test data was obtained by the curve represented by Legend 5, where elasto-plastic rebars and the steel section were used. The load–displacement relationship made by Legend 7 was obtained when the concrete was modeled based on the confined Mander constitutive equations. The flexural strengths of the curves corresponding to Legends of 2 and 3 were predicted to be quite large in the region where the test data were descending. In these curves, the rebar and steel sections were modeled to be embedded in concrete and buckling or necking of the rebars and steel section was not allowed. The tie model demonstrated a better correlation with the test than the estimates produced using the embedded model. The flexural strength of the curve represented by Legend 2 was obtained with a base, while the other specimens did not have bases. The unconfined Kent–Park constitutive curves, shown with Legends 4 to 6, were extended to a strain of 0.01.

## 3. Results and Discussion

### 3.1. Verification of the Analytical Model with Finite Element Analysis Results

**Figure 11:**
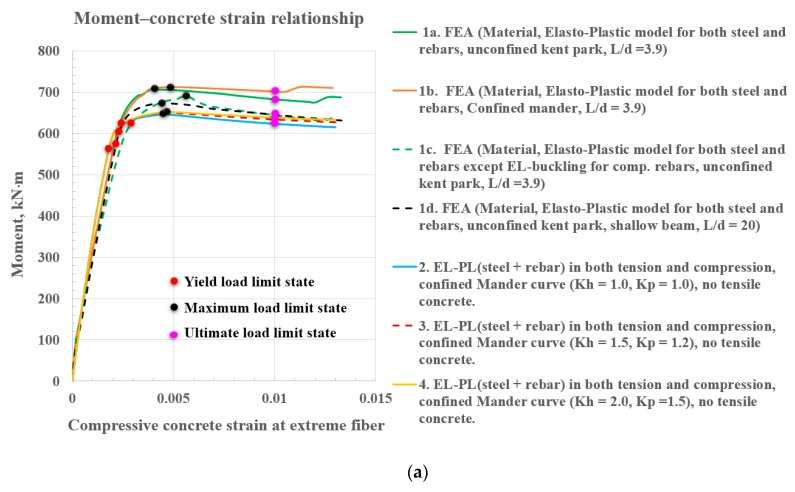

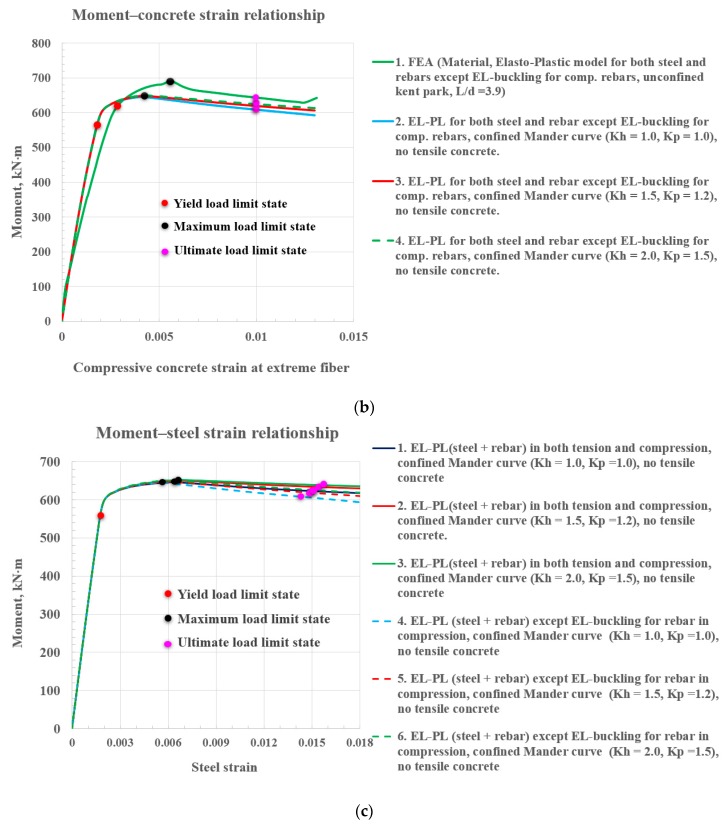
Parameters influencing the flexural strength of the steel section encased in structural concrete; finite element analysis compared with test data; moment–compressive concrete strain relationship based on tie models with fixed base (with base vs. without base): (**a**) Moment–compressive concrete strain relationship; (**b**) moment–compressive concrete strain relationship with buckling for rebar in compression; (**c**) moment–tensile steel strain relationship.

The post-yield behavior of the precast composite beams was obtained based on the iterated strain compatibility approach using Equations (3)–(27). The analytically calculated flexural strengths (indicated Legends 1a to 1d of Figure 11a) was compared with the results of the FEA study (indicated Legends 2 to 4 of Figure 11a) at yield, maximum load, and ultimate load limit states in Figure 11a,b. The shallow beam influence of the confined concrete effect by the steel section for the various confining factors (K_h_ and K_p_) was also shown with moment–strain relationships represented by Legends 2, 3, and 4 in Figure 11a. The moment–strain relationship (refer to Legend 4 in Figure 11a) of the shallow beam based on an analytical model with confining factors of K_h_ with 2.0 and K_p_ with 1.5 were compared with FEA data (refer to Legend 1a in Figure 11a for the deep beam with a L/d ratio of 3.9) by errors of 6.58% (indicated by red dots) and 7.45% (indicated by black dots) at the yield and maximum load limit state, respectively. One of reasons for the difference was the deep beam effect. A significant amount of the load was carried to the supports by a compression force combining the load and the reaction in deep beams, resulting in significant shear deformations compared to pure flexure caused by a non-linear strain distribution. The stress distribution is not linear even in the elastic stage, and the shape of the concrete compressive stress block may not be parabolic at the ultimate limit state. Greater differences were observed for the maximum load limit state than in the yield limit state for deep beams with a L/d ratio of 3.9, indicating that the influence of the plasticity of concrete on the post-yield deformation of the composite beams at the maximum load limit state was more significant than that at the yield limit state. The difference between analytical and numerical models at the maximum load limit state was part of the reason for ignoring the plastic rotation due to the inelastic energy dissipation in Equations (3)–(28) and (A1)–(A8). The analytical estimation of curvatures in sections based on strain compatibility did not consider inelastic energy dissipation. An accurate prediction of the post-yield deformation of the composite beams at a maximum load limit state must account for the plastic rotations of the sections between cracks, reflecting the inelastic energy dissipation associated with diagonal concrete cracks. The stiffening effect of concrete tension between cracks and plastic strains occurring in the steel section should be included as well. However, the flexural capacity obtained by an analytical model with confining factors of K_h_ (2.0) and K_p_ (1.5) (refer to Legend 4 in Figure 2; Figure 11a) was closer to that predicted by the numerical moment–strain relationship (refer to Legend 1d in Figure 11a) for the 9 m shallow beam with a L/d ratio of 20. These differences reduced to 4.68% and 2.93% at yield and the maximum load limit state, respectively. It is worth noting that the moment–strain relationship represented by Legend 2 demonstrates a greater discrepancy when the confining effect offered by steel sections was not included in the analysis in which K_h_ and K_p_ was implemented as 1.

### 3.2. Influence of Buckling Effect of Reinforcing Steels on the Flexural Strength

In Figure 11b,c, the influence of the buckling effect of reinforcing steels in compression on the flexural strength of the composite section was explored for varied compressive concrete strain and tensile steel strain, respectively. Figure 11c demonstrates a moment–steel strain relationship of all the analyses shown in Figure 11a,b. The flexural strength of the composite section with buckling of reinforcing steels in compression decreased more rapidly than one that did not consider buckling of reinforcing steels in compression without considering the confinement effect provided by the steel section encased in concrete. 

Legends 4, 5, and 6 of Figure 11c show the rapid degradation of the flexural strength of the composite section with buckling of reinforcing steels in compression. However, the decrease of flexural strength in the composite beams considering confining effects offered by the steel section became retarded when ignoring compressive buckling failure as shown in Legends 1, 2, and 3. When compressive buckling failure was not considered, the compressive concrete area with highly and partially confined zones at the yield and maximum load limit state were small, preventing the confining factors from significantly influencing the flexural load resisting capacity. Rebars should be modeled with buckling in the compression zone to accurately predict flexural capacity of composite beams. Table 1 summarizes moments at the maximum load and design load limit states, which were identified from Figure 11a,b. The design load limit state is defined at a concrete strain corresponding to 0.003.

## 4. Conclusions

(1) Significant implications of the information presented in the body of the study

Understanding the post-yield structural behavior of composite beams composed of structural steel and concrete is quite a complicated issue. One of the most fundamental requirements in predicting an accurate post-yield behavior of composite beams was to understand the constitutive relationships of concrete with all the confinements provided by the surrounding structural elements such as stirrups and the wide flanges of steel sections. Simplified but accurate analytical equations based on iterated strain compatibility were proposed to estimate the equilibrium neutral axis and flexural moment strength reflecting the buckling of reinforcing rebar in the compression zone. The post-yield behavior of composite beams was accurately estimated, considering confining effects on the concrete by shear reinforcement and the steel flange. A numerical investigation of the composite beams using finite element analysis (FEA) based on damaged concrete plasticity was also presented with nonlinear parameters to verify the analytically predicted post-yield behavior with the two confining effects. In this study, the moment–strain relationships for different confining factors were obtained by an analytical model based on strain compatibility. The influence of confinement in concrete caused by rebar and steel on the post-yield behavior of composite beams was explored. 

(2) Research impact both within and beyond academia

For deep beams with an L/d ratio of 3.9, inconsistencies at the yield limit state were demonstrated between the flexural strengths calculated based on strain compatibility and those obtained by the nonlinear finite element investigation, indicating that the flexural strength of deep beams was not accurately estimated when only the curvature of the sections based on strain compatibility was considered. However, the flexural capacities obtained by a nonlinear FEA based on the 9 m shallow beam with a L/d ratio of 20 were relatively well compared with the analytical estimates based on strain compatibility both at yield and maximum load limit states, respectively, when the effects of deep beam and concrete plasticity were minimized. The deep beam effect was one of the reasons for the difference in the prediction of the post-yield deformations and the flexural capacities of composite beams at the maximum load limit state because the analytical estimation based on strain compatibility cannot consider the plastic rotation due to inelastic energy dissipation.

(3) Influence of the steel sections on the flexural capacities of the concrete-encased steel beams

Studies that focused on the consideration of double confinements (provided by both transverse reinforcements and a wide flange steel section) are absent from the literature to some extent in conventional composite beam analysis, ignoring the contribution of confined concrete by steel sections. The highly and partially confined zones for compressive concrete sections at the yield and maximum load limit state were limited, being unable to fully activate the confining effects on the flexural load resisting capacity. However, the changes of flexural load resisting capacity were observed due to the confining effects by the steel section when compressive buckling failure was considered. When considering confining effects by the steel section, a decrease in moment strengths was observed when rebars buckled during compression. However, the retarded decrease in moment strengths was observed from the beam when compressive buckling failure was not considered. Rebar buckling in the compression zone is not frequently considered for the flexural analysis of beams. However, it was shown that an accurate flexural capacity of composite beams can be obtained when rebar was modeled with buckling in the compression zone. The analytical algorithm based on strain compatibility developed in this study can be extended to understand the post-yield behavior of columns with axial loads.

## Figures and Tables

**Table 1:** Flexural capacities at the maximum load and design load limit states.

	K_p_	K_h_	Maximum Load/Concrete Strain	Maximum Moment/Concrete Strain	Design Load (Concrete Strain = 0.003)	Design Moment (Concrete Strain = 0.003)
**Elasto-Plastic (Steel + Rebar) in Both Tension and Compression, Confined Mander Curve, Figure 11a**						
Legend 2	1.0	1.0	380.6 kN/0.0046	647.0 kN∙m/0.0046	373.0 kN	634.1 kN∙m
Legend 3	1.2	1.5	382.7 kN/0.0046	650.5 kN∙m/0.0046	373.5 kN	634.9 kN∙m
Legend 4	1.5	2.0	384.1 kN/0.0046	652.9 kN∙m/0.0046	374.3 kN	636.3 kN∙m
**Elasto-Plastic (Steel + Rebar) in both Tension and Compression except EL-Buckling for Rebar in Compression, Confined Mander Curve, Figure 11b**						
Legend 2	1.0	1.0	379.7 kN/0.0042	645.5 kN∙m/0.0042	373.5 kN	635.0 kN∙m
Legend 3	1.2	1.5	381.5 kN/0.0042	648.5 kN∙m/0.0042	374.1 kN	635.9 kN∙m
Legend 4	1.5	2.0	382.8 kN/0.0042	650.8 kN∙m/0.0042	374.9 kN	637.3 kN∙m

## Nomenclature

*A_ri_*	Area of rebar layer *i* (*i* = 1,2), mm^2^
*A_si_*	Area of part i of H-steel section, mm^2^
*B*	Width of the beam section, mm
*B_i_*	Width of unconfined, confined, partially, highly confined concrete area (*i* = 1–4), mm
*c_i_*	Height of concrete compression zone of unconfined, confined, partially, highly confined concrete area (*i* = 1–4), mm
*c*_11_, *c*_12_	Height of components of unconfined area, mm
*c*_21_, *c*_22_	Height of components of unconfined area inside, mm
*C_ci_*	Compressive force given by unconfined, confined, partially, highly confined concrete area, kN
*C’_ci_*	Compressive force given by unconfined, confined, partially, highly confined concrete area inside, kN
*C*_*c*11_, *C*_*c*12_	Components of compressive force given by unconfined area, kN
*C*’_*c*11_, *C*’_*c*12_	Components of compressive force given by unconfined area inside, kN
*D*	Depth of the beam section, mm
*d_i_*	Distance from rebar layer *i* (*i* = 1,2) to bottom of beam, mm
*d_c_*	Distance from centroid to bottom of the beam, mm
*d_s_*	Distance from bottom flange of H-steel to bottom of the beam, mm
*E_s_*	Young’s modulus of steel, MPa
*E_r_*	Young’s modulus of rebar, MPa
$F_{axial}$	The external axial forces, kN
$F_{R_{tension}}$	The internal forces contributed by rebar in tension, kN
$F_{R_{compression}}$	The internal forces contributed by rebar in compression, kN
$F_{steel_{tenstion}}$	The internal forces contributed by steel in tension, kN
$F_{steel_{compression}}$	The internal forces contributed by steel in compression, kN
*F_ri_*	Force given by rebar layer *i* (*i* = 1,2), kN
*F_si_*	Force given by part *i* of L-steel section, kN
*ε_cmi_*	Strain at fiber of unconfined, confined, partially, highly confined concrete area (*i* = 1–4)
*ε_yR_*	Yield strain of rebar
*ε_yS_*	Yield strain of steel
*ε_ri_*	Strain of rebar layer *i* (*i* = 1, 2)
*ε_si_*	Strain respect to part *i* of H-steel section
*f_yR_*	Yield strength of rebar, MPa
*f_yS_*	Yield strength of steel, MPa
*f '_c_*	Compressive strength of unconfined concrete, MPa
*f '_cc_*	Compressive strength of confined concrete, MPa
*f*_*c*1_	Concrete compressive stress in term of concrete strain of unconfined area, MPa
*f*_*c*2_	Concrete compressive stress in term of concrete strain of confined area, MPa
$f_{\varepsilon_{cm1}}$	Concrete compressive strength at extreme fiber of unconfined region, MPa
$f_{\varepsilon_{cm2}}$	Concrete compressive strength at extreme fiber of confined region, MPa
*h*	Depth of H-steel section, mm
K_h_	Confinement factors for highly confined concrete
K_p_	Confinement factors for partially confined concrete
*t*_*f*1_	Top flange thickness of H-steel section, mm
*t*_*f*2_	Bottom flange thickness of H-steel section, mm
*t_w_*	Web thickness of H-steel section, mm
*x_i_*	Distance from the edge of the concrete confined areas to the bottom of the beam (*i* = 1–3), mm
*w*	Width of H-steel section, mm
*α_i_*	Stress factors for the concrete areas *i*
*α’_i_*	Stress factors for the concrete areas inside *i*
$\sigma_{t0}$	Uniaxial tensile stress, MPa
*γ_i_*	Centroid factor for the concrete areas *i*
*γ’_i_*	Centroid factor for the concrete areas inside *i*
ϵ	Eccentricity
*f*_*b*0_	Initial equibiaxial compressive yield stress of concrete, MPa
*f*_*c*0_	Initial uniaxial compressive yield stress of concrete, MPa
*K*	The ratio of the second stress invariant on the tensile meridian
G(σ)	Non-associated plastic flow potential, Druker-Prager formulation
$\bar{p}$, $\bar{q}$	The plane in which plastic potential function is defined.
ψ	Dilation angle

## Appendix A

The compressive concrete blocks $c_{2},\;c_{3},\;c_{4}:$

$c_{2}=c_{1}-x_{1};\;x_{1}=40\;\mathrm{mm}$
$c_{3}=c_{1}-x_{2};\;x_{2}=93.75\;\mathrm{mm}$
$c_{4}=c_{1}-x_{3};\;x_{3}=139\;\mathrm{mm}$

The mean stress factors *α* for the four zones are obtained by

(A1) $$\alpha_{1}=\;\frac{\int_{0}^{0.002}f_{c1}d\varepsilon_{c}}{0.002f_{c}^{\prime }}$$

(A2) $$\alpha \prime_{1}=\;\frac{\int_{0}^{0.002}f_{c1}d\varepsilon_{c}}{0.002f_{c}^{\prime }}$$

(A3) $$\alpha_{2}=\;\frac{\int_{0}^{\varepsilon_{cm2}}f_{c2}d\varepsilon_{c}}{f\prime_{cc}\varepsilon_{cm2}};\;\varepsilon_{cm2}=\varepsilon_{yS}\times \frac{c_{2}}{d_{s}+h-c_{1}}$$

(A4) $$\alpha \prime_{2}=\;\frac{\int_{0}^{\varepsilon_{cm3}}f_{c2}d\varepsilon_{c}}{f{’}_{cc}\varepsilon_{cm3}}$$

(A5) $$\alpha_{3}=\;\frac{\int_{0}^{\varepsilon_{cm3}}K_{p}f_{c2}d\varepsilon_{c}}{K_{p}f{’}_{cc}\varepsilon_{cm3}}=\;\frac{\int_{0}^{\varepsilon_{cm3}}f_{c2}d\varepsilon_{c}}{f{’}_{cc}\varepsilon_{cm3}};\;\varepsilon_{cm3}=\varepsilon_{yS}\times \frac{c_{3}}{d_{s}+h-c_{1}}$$

(A6) $$\alpha \prime_{3}=\;\frac{\int_{0}^{\varepsilon_{cm4}}K_{p}f_{c2}d\varepsilon_{c}}{K_{p}f{’}_{cc}\varepsilon_{cm4}}=\;\frac{\int_{0}^{\varepsilon_{cm4}}f_{c2}d\varepsilon_{c}}{f{’}_{cc}\varepsilon_{cm4}}$$

(A7) $$\alpha_{4}=\;\frac{\int_{0}^{\varepsilon_{cm4}}K_{h}f_{c2}d\varepsilon_{c}}{K_{h}f\prime_{cc}\varepsilon_{cm4}}=\frac{\int_{0}^{\varepsilon_{cm4}}f_{c2}d\varepsilon_{c}}{f\prime_{cc}\varepsilon_{cm4}};\;\varepsilon_{cm4}=\varepsilon_{yS}\times \frac{c_{4}}{d_{s}+h-c_{1}}.$$

Here, the centroid factors γ are given as

(A8) $$\gamma_{1}=1-\frac{\int_{0}^{0.002}\varepsilon_{c}f_{c1}d\varepsilon_{c}}{0.002\int_{0}^{0.002}f_{c1}d\varepsilon_{c}}$$

(A9) $$\gamma \prime_{1}=1-\frac{\int_{0}^{0.002}\varepsilon_{c}f_{c1}d\varepsilon_{c}}{0.002\int_{0}^{0.002}f_{c1}d\varepsilon_{c}}$$

(A10) $$\gamma_{2}=1-\frac{\int_{0}^{\varepsilon_{cm2}}\varepsilon_{c}f_{c2}d\varepsilon_{c}}{\varepsilon_{cm2}\int_{0}^{\varepsilon_{cm2}}f_{c2}d\varepsilon_{c}}$$

(A11) $$\gamma \prime_{2}=1-\frac{\int_{0}^{\varepsilon_{cm3}}\varepsilon_{c}f_{c2}d\varepsilon_{c}}{\varepsilon_{cm3}\int_{0}^{\varepsilon_{cm3}}f_{c2}d\varepsilon_{c}}$$

(A12) $$\gamma_{3}=1-\frac{\int_{0}^{\varepsilon_{cm3}}\varepsilon_{c}\left(K_{p}f_{c2}\right)d\varepsilon_{c}}{\varepsilon_{cm3}\int_{0}^{\varepsilon_{cm3}}(K_{p}f_{c2})d\varepsilon_{c}}=1-\frac{\int_{0}^{\varepsilon_{cm3}}\varepsilon_{c}f_{c2}d\varepsilon_{c}}{\varepsilon_{cm3}\int_{0}^{\varepsilon_{cm3}}f_{c2}d\varepsilon_{c}}$$

(A13) $$\gamma \prime_{3}=1-\frac{\int_{0}^{\varepsilon_{cm4}}\varepsilon_{c}\left(K_{p}f_{c2}\right)d\varepsilon_{c}}{\varepsilon_{cm4}\int_{0}^{\varepsilon_{cm4}}\left(K_{p}f_{c2}\right)d\varepsilon_{c}}=1-\frac{\int_{0}^{\varepsilon_{cm4}}\varepsilon_{c}f_{c2}d\varepsilon_{c}}{\varepsilon_{cm4}\int_{0}^{\varepsilon_{cm4}}f_{c2}d\varepsilon_{c}}$$

(A14) $$\gamma_{4}=1-\frac{\int_{0}^{\varepsilon_{cm4}}\varepsilon_{c}\left(K_{h}f_{c2}\right)d\varepsilon_{c}}{\varepsilon_{cm4}\int_{0}^{\varepsilon_{cm4}}\left(K_{h}f_{c2}\right)d\varepsilon_{c}}=1-\frac{\int_{0}^{\varepsilon_{cm4}}\varepsilon_{c}f_{c2}d\varepsilon_{c}}{\varepsilon_{cm4}\int_{0}^{\varepsilon_{cm4}}f_{c2}d\varepsilon_{c}}.$$
